# Examining the existing usage gap of electronic health records in the United States: A study of National Health Survey

**DOI:** 10.1016/j.ssmph.2023.101577

**Published:** 2023-12-10

**Authors:** Qinglin Hu, Yuhui Yao, Jing Han, Xin (Thomas) Yang, Jason Parton

**Affiliations:** a Institute of Data and Analytics, The University of Alabama, Tuscaloosa, AL, 35487, USA; b Department of Community Medicine and Population Health, The University of Alabama, Tuscaloosa, AL, 35487, USA; c Department of Management, Culverhouse College of Business, The University of Alabama, Tuscaloosa, AL, 35487, USA; d Department of Information Systems, Statistics, and Management Science, Culverhouse College of Business, The University of Alabama, Tuscaloosa, AL, 35487, USA

**Keywords:** Electronic health records, Health disparities, Underserved population, Geographical variations, Health management

## Abstract

**Background:**

Little is known about the extent of geographic variation in online health record usage and related demographic characteristics in the United States.

**Methods:**

In order to examine geographical variation in the usage of online e-health records (EHR) patient portals in the US, and the sociodemographic factors effects on the access and use of the EHR patient's portal. This study using data from the 2019 and 2020 Health Information National Trends Survey. Specifically, predictors associated with accessing patients' EHR portal were examined. Furthermore, geographic variation of EHR portal’ availability and usage gap were examined and mapped.

**Results:**

Respondents had significantly higher likelihood to access EHR portals when they are higher educated, willing to seek health information online, insured and had regular providers (adjusted OR = 2.01, 95% CI: 1.44 – 2.80; adjusted OR = 3.51, 95% CI: 2.49 – 4.94; adjusted OR = 2.38, 95% CI: 1.05 – 5.43; adjusted OR = 2.1, 95% CI: 1.51 – 2.92, respectively). Individuals living in Central-West, South regions or other non-urban areas as well as deprived urban areas are less likely to access their EHR portals (adjusted OR = 0.6, 95% CI: 0.41 – 0.89). Furthermore, we found that people living in the Midwest, Southern regions, and Mountain rural areas are more likely to have greater difficulties to access EHR than other regions. Therefore, populations residing in these underserved (deprived urban, rural or remote) areas tend to face more considerable obstacles to e-healthcare.

**Conclusions:**

Improve the disparities, accessibility, and educational initiatives on the usage of eHealth resources and encouragement from both healthcare providers and policymakers should be implemented with a particular focus on targeting non-urban areas and underserved population.

## Highlights

• This study observes the usage gap between EHR adoption and access for patients through both geographical and statistical methods.
• People living in the Midwest, Southern, and Mountain areas exhibit a higher propensity for encountering heightened challenges in accessing EHR.
• Female, uninsured, less educated, no regular provider, living in rural areas or deprived urban areas are key barriers for accessing EHR.
• The mixed method provides a unique and innovative way for health professionals and policy maker to mitigate health disparity in various domains.

## 1 Introduction

In its recent report, the Healthy People 2030, the Office of Disease Prevention and Health Promotion (ODPHP) in the Department of Health and Human Services recommended that one of the objectives of health communication and health information technology is to “increase the proportion of adults offered online access to their medical record” as their baseline objective. The Healthy People 2030 report also indicates an “increase in the proportion of adults who use IT to track health care data or communicate with providers”, such as care received, test results, or upcoming medical appointments (Office of Disease Prevention and Health Promotion, 2020). In recent times, studies have described the capabilities of the use of electronic health records (EHR) as a critical tool to improve population health outcomes and achieve health equity. Numerous studies reported a strong association between electronic access to health records and improved health self-management, attitudes, knowledge, and behavior among patients (Office of Disease Prevention and Health Promotion, 2020; U.S. Department of Health and Human Services; Baker et al., 2003; Czaja, Manfredi, & Price, 2003; Lustria, Smith, & Hinnant, 2011).

Following the implementation of the Health Information Technology for Economic and Clinical Health Act (HITECH) in 2009, the EHR was widely adopted by most healthcare providers (Goldzweig, Orshansky, & Paige, 2013; Ralston, Rutter, & Carrell, 2009a). In 2009, about 5% of hospitals in the US utilized a web-based EHR system for core healthcare functions including health information and data, immediate access to key information, such as patients' diagnoses, allergies, lab test results, and medications (Kruse et al., 2015). Further, the EHR system facilitated dissemination of reliable prevention and treatment information to patients, offered providers a platform for patient health coaching and strengthen the patient and provider relationship (Byczkowski, Munafo, & Britto, 2014; Mayer et al., 2007; Nijland et al., 2011; Osborn et al., 2010). By 2021, approximately 96% of US hospitals have possession of an EHR certified by HHS, including laboratory test results, upcoming appointments, summaries of past visits, and secure communication with health care providers (Office of the National Coordinator for Health Information Technology, 2022).

Access to electronic health (eHealth) information technologies makes it easier for patients to engage in their healthcare. For instance, electronic access to health information and personal health records allows patients to have active involvement in their own care (Delbanco et al., 2012; Ralston et al., 2004; Reti et al., 2010). Patients are more active in managing and monitoring their health conditions through patient portals (Or & Tao, 2014; Ricciardi et al., 2013); more likely to communicate with providers across multiple medical facilities about their health records, lab results and request for medication refills; and more motivated to learn new information about their diseases (Emont, 2011; Goldzweig et al., 2013; Ralston et al., 2009b; Sarkar et al., 2010). Patients also desire to monitor their health by entering their daily blood sugar levels or weight, exercise, other health indicators, and symptoms, which gives patients a better way to manage their health conditions (Goldzweig et al., 2013; Ralston et al., 2009b). Other research suggests that an eHealth approach - electronic processes and communication features adopted in patient portals - improved patient empowerment and physical activity, which subsequently improved their health status and quality of life and reduced their need for supportive care (Kuijpers et al., 2013).

Despite the widespread availability and benefits of EHR systems, only three in 10 of US healthcare patients use their online health management portals in 2020. Furthermore, the proportion of patients who were offered and accessed a patient portal did not increase between 2019 and 2020 (Johnson, Richwine, & Patel, 2020). While previous studies provided essential findings of the patients who use EHR portals to access their health record (Ammenwerth et al., 2017; Pell et al., 2015; Pyper et al., 2002), few studies examined which groups have barriers to access demographically and spatially, and the causes behind those situations. Prior works reveal that disparities in health care access exist among different demographic groups, especially for low income, the minority (e.g., Black, and Hispanic or Latino patients), and disability populations (Andrulis, 1998; Betancourt et al., 2003; Krahn, Hammond, & Turner, 2006). It is unclear whether those population groups are still experiencing difficulties in accessing their EHR portals or not. The EHR portals have been described as a useful tool to convey health-related information (Office of Disease Prevention and Health Promotion, 2020; U.S. Department of Health and Human Services; Baker et al., 2003; Czaja et al., 2003; Lustria et al., 2011), enhance better understanding and control patient's health conditions (Delbanco et al., 2012), and facilitate communication between patients and providers (Ralston et al., 2009b; Reti et al., 2010). It is also expected to be an alternative way to provide health care service to those who have difficulties getting to the regular health care visit and may finally improve health disparities.

Numerous studies suggest that disparities impact the extents of patients accessing EHR portals. Most of the studies focused on the socio-economic implications for accessing patient portals. Hing and Burt (2009) used the National Ambulatory Medical Care Survey and the National Hospital Ambulatory Medical Care Survey data to show that uninsured Black and Hispanic or Latino patients were less likely to use patient portals, compared with privately-insured White patients (Hing & Burt, 2009). In the Yeager et al. (2010) study, they found that although EHR adoption has improved in recent decades, doctors who serve an elderly population are still less likely to adopt EHR system (Yeager, Menachemi, & Brooks, 2010). Another study shows that low-income patients, especially Medicaid patients, tend to have lower odds of using their patient portals (Coffman et al., 2014). Other studies also found that females and people with higher levels of education were more associated with the registration with EHR systems (Munir & Boaden, 2001; Roblin et al., 2009). If certain population groups have more difficulties accessing online portals, the use of online portals in more disadvantaged groups may contribute to health disparities.

Regional variations in healthcare experiences, health system performances, and health care access have been repeatedly documented. Unfortunately, there is limited works on the knowledge of the use and access to EHR system geographically. It is also important to understand the concurrent social determinants of health that could impact on the access and use of the EHR system. More so, understanding the geographical variation in the use of EHR portals could provide us a fuller picture of the current disparities impacting patient access to EHR systems, such as people living in underserved or deprived areas.

In this paper, we examine geographical variation in the use of EHR patient portal and the effects of socio-demographic factors associated with patients’ portal use. Moreover, this study uses correlation maps to visualize the spatial associations between impacting demographic factors and the use of 10.13039/100000081EHR patient portals, along with the evidential support derived from instances obtained through the statistical analysis. This research offers a timely and thorough evaluation of the contemporary utilization of Electronic Health Records (EHR) within the United States.

## 2 Data acquisition and methodology

### 2.1 Sample population

We conducted data extraction from the Health Information National Trends Survey (HINTS) for two survey iterations in 2019 (HINTS 5 cycle 3) and 2020 (HINTS 5 cycle 4). HINTS is a nationally representative household interview cross-sectional survey of US adults aged ≥18 years who are noninstitutionalized civilians. The sampling frame consisted of a two-stage design where the first stage involved selecting a random sample of addresses from a database of residential addresses, and the second stage one adult was selected within each household. Our target sample was participants with valid responses on the access and use of their online EHR portal. A total of 9303 respondents were included for descriptive analyses, and an analytic sample of 5235 with complete responses for all measures was used in the logistic regression model. The survey methodology can be viewed and downloaded from the National Cancer Institute (NCI) Health Information National Trends Survey (HINTS) website (Health Information National Trends Survey). The HINTS data was granted exempted status by the Internal Review Board (IRB) of the NCI's Office of Human Subjects Research and by the IRB of the organization that administers the survey, Westat. The current analysis was exempted from review by the IRB of the University of Alabama. The data used in this analysis with no respondent identifiers.

### 2.2 Measures

Our variables of interest are two questions that assess the EHR portal adoption and access. For addressing the adoption and usage of the EHR, the questions of ‘Have you ever been offered online access to your medical records by your health care provider?’ and ‘How many times did you access your online medical record in the last 12 months?’ were employed. Given the former question with a response that the patients were offered and access the online portals, a new outcome is dichotomized using the latter question. A value of ‘0’ represents respondents have accessed and used the offered EHR portals and ‘1’ represents a value if respondents haven't had a chance to access and use the offered EHR portals.

### 2.3 Sociodemographic Variables

The following socio-demographics were included in our descriptive and logistic regression analyses: gender (male, female), age group (18–24, 25–44, 45–64, 65–74, 75+), education (less than high school or completed high school, some college, college graduate or higher), race/ethnicity (Non-Hispanic White, Non-Hispanic Black, Hispanic, Asian and other), urbanity (urban, non-urban determined by Rural-Urban Continuum codes), household income (<$20,000, $20,000 - $50,000, $50,000 - $75,000, >$75,000).

### 2.4 Health-related variables

Health information-seeking behavior (HISB) was assessed by the question ‘In the past 12 months have you used a computer, smart phone, or other electronic means to look for health or medical information for yourself?’ If respondents answered ‘yes’, we coded HISB as ‘1’. Otherwise, we coded HISB as ‘0’. General health was categorized as excellent/very good (3), good (2), fair/poor (1). Whether respondents have health care coverage and regular providers (1, 0) were also included in the analyses.

### 2.5 Geographic location

States code for each respondent was obtained through the sampling process. We excluded any respondent with a state code for Alaska and Hawaii due to the scarcity of samples.

### 2.6 Data analysis

Descriptive statistics such as frequency and prevalence by subgroups (gender, education, etc.) were calculated for the total sample as well as two subgroups defined by adoption by providers and access by patients. Chi-square tests were used to test the bivariate association between outcome and measures in descriptive statistics. To assess the association between outcome and measures adjusted by other covariates, we modeled the respondents with at least one-time access EHR portal using multivariable logistic regression, including all measures to identify factors that independently associated with accessing EHR behavior. In order to obtain estimates that are nationally representative, we used sample weights from survey data for population estimates through the jackknife replication method. A significance level of 0.05 is considered statistically significant. All analyses were conducted using SAS 9.4 (SAS Cary, NC).

**Fig. 1:**
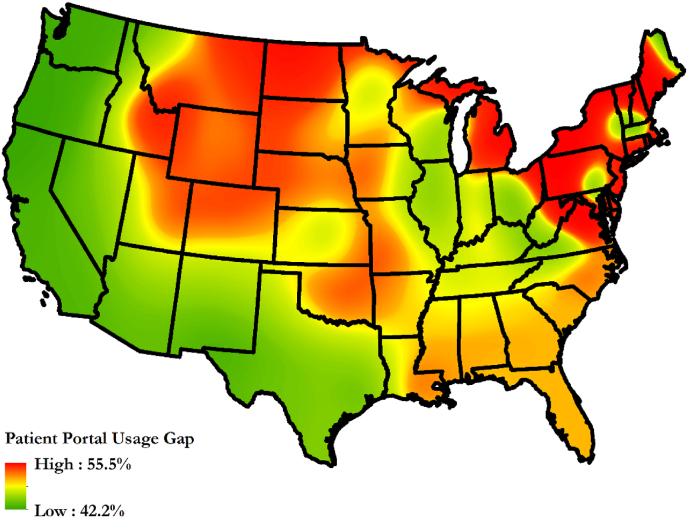
Geographic distribution of usage gap of EHR patient portals between actual adopted and actual use in the U.S.

**Fig. 2:**
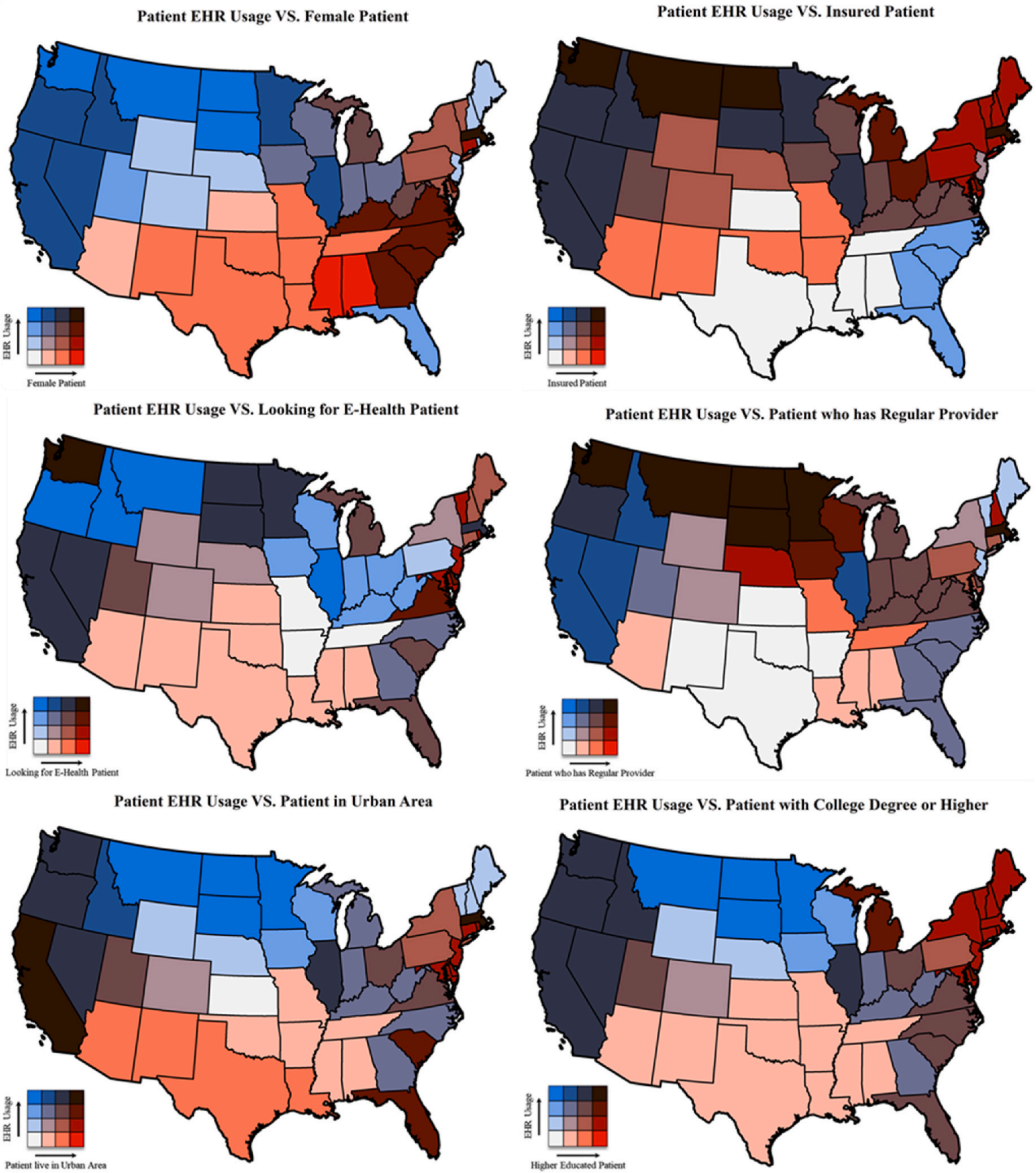
Geographic distribution of association between online medical record usage and significant demographic and health-related factors.

Geographic distribution on the state-level was mapped using the weighted prevalence of the outcome and independent variables. A geographic information map was generated to examine the geographic variation of patient portal adoption and access gap in the United States (see Fig. 1). EHR portal use gap was defined as the weighted prevalence difference between individuals who were offered portal and who were access portal in each state. The spline method is employed in this study as an interpolation method that estimates values using a mathematical function that minimizes overall surface curvature, which generates a smooth surface constrained by the sample data range (Kaw, Kalu, & Besterfield, 2008; Rutten et al., 2012). This method provides a better way to ensure the stability and precision of calculations with the large sample size. Moreover, bivariate choropleth maps (see Fig. 2) were generated to explore the geographic distribution patterns of the association for both the outcome and its associated factors. Bivariate choropleth mapping is a technique for concurrently representing categories of values associated with two variables on a map. It visually conveys the relationship between two related variables with distinct symbols and colors. This method provides a straightforward and precise means of visually depicting the spatial distribution and mutual interaction of the two variables. Additionally, it allows for a straightforward evaluation of how these two attributes vary in relation to one another (Biesecker, Zahnd, Brandt, Adams, & Eberth, 2020).

## 3 Results

Table 1 shows raw sample frequency and weighted percentages for sociodemographic characteristics and health-related measures for the total sample and by access to EHR portals. Respondents who had access to online EHR portals were likely to be female (57.0%), and have household income higher than $50,000 (66.9%). People who access their EHR portals were also much likely to look for health information online (81.6%), having health insurance (95.3%) and a regular provider (74.5%).

Table 2 illustrates global tests comparing the intercept-only model with the model utilizing the input from Table 1. These tests demonstrate the enhancement achieved by incorporating sociodemographic characteristics and health-related variables (p < .001). That is, at least one variable of the input correlates with the outcome of the use of online EHR portals significantly. The results in the table present compelling statistical evidence of the observed improvement.

Fig. 1 shows the spatial pattern of the usage gap between who were offered portal and who were access portal. The geographic variability in this usage gap is larger in the Midwest and Northeastern States. The gaps between the portals being offered and the portals being used were relatively narrower in the Southeastern States. Interestingly, the EHR are being widely offered and used for patients in West Coast areas (California, Washington, Oregon and Nevada). The similar pattern extended to Southwestern States, such as Arizona and Texas. The East Coast States are also present a high proportion of people using offered EHR portals.

Table 3 indicates the associations of sociodemographic characteristics and health-related variables with the outcome. Crude odds ratios (OR) and 95% confidence intervals (CI) were obtained by modeling the probability of respondents who had access to online medical records using a set of bivariate logistic regression models with one independent variable at one time. Most of the factors were significantly associated with access to online medical records, except for patients’ gender and general health condition. Adjusted odds ratios and its 95% CI were assessed by multivariable logistic regression models with all the measures included, leading to attenuation of association of gender, age group, race, household income and general health with the outcome. Consistent with descriptive analysis in table 1, respondents who live in non-urban areas had lower odds of accessing online records (adjusted OR = 0.60; 95% CI: 0.41 – 0.89). Education level was positively associated with accessing online records (p < .001), and respondents with college graduate or higher degree had roughly 100% more odds to access online records than those without a college degree (adjusted OR = 2.01, 95% CI: 1.44-2.80). Moreover, people who looked for health information online were more likely to access their online records (adjusted OR = 3.51; 95% CI: 2.49–4.94). Similarly, respondents who had health insurance and regular providers had significantly higher odds of accessing online records compared with those without health insurance and regular providers (adjusted OR = 2.38; 95% CI: 1.05 – 5.43 and adjusted OR = 2.1; 95% CI: 1.51 – 2.92 respectively).

To facilitate the practical application of the adjusted logistic regression model, we provide Eq. (1) for estimating an individual outcome entry with the significant associations in Table 3, as follows

Eq. 1 $$\log\left(OR\right)=\log\left(\frac{\hat{\pi }}{1-\hat{\pi }}\right)=intercept+0.70\left(\text{College}\;\text{graduate}\;\text{or}\;\text{higher}\right)+0.39\left(\text{Some}\;\text{college}\right)-0.51\left(\text{Non}-\text{urban}\right)+1.26\left(\text{Look}\;\text{for}\;\text{health}\;\text{info}.\right)+0.87\left(\text{Has}\;\text{health}\;\text{insurance}\right)+0.74\left(\text{Has}\;\text{regular}\;\text{Provider}\right)$$

where $\hat{\pi }$ is the fitted value and intercept is −2.5962.

Key indicators from our logistic regression model were identified to demonstrate their potential significant impact in preventing patients from accessing the patient portals, considering a comprehensive view of multiple factors. The factors, with the associated outcome variable, were mapping in Fig. 2 respectively. Each map illustrates the bivariate association between impact factor and the EHR usage at state level. The utilization of a bivariate color palette in the maps, characterized by a gradient that transitions from lighter to darker shades in two distinct directions, conveys a meaningful message regarding the association between two variables. In this representation, the gradual shift in color intensity serves as a visual indicator of the progressive convergence or divergence of the variables under consideration. The lighter hues, denoting lower association, represent areas where the two variables exhibit limited mutual influence, while the darker hues signify a stronger association in terms of EHR (Electronic Health Record) usage and the target factor within the state. This approach enhances our understanding of the spatial relationship between these variables, offering a visually intuitive means to discern the degree of proximity or disparity. For example, in Fig. 2, female, uninsured, less educated, lack of E-health resource, no regular provider, no insurance or living in rural areas or deprived urban areas, patients either in one of these five characteristic categories and locate in West North Central division or South regions are more likely having barriers to access their patient portals than other distinctive population groups. Furthermore, we also found that (1) high proportion of female use their EHR in East Coast than other regions; (2) West Pacific or New England divisions have more insured and educated population that may due to the socio-economic characteristics of those states; (3) compare with South regions, people in Midwest regions tend to have regular providers and regular health care visit; (4) Those who live in urban areas in either West Coast or East Coast areas were more propensity to use their patient portals than people in other areas.

**Fig. 3:**
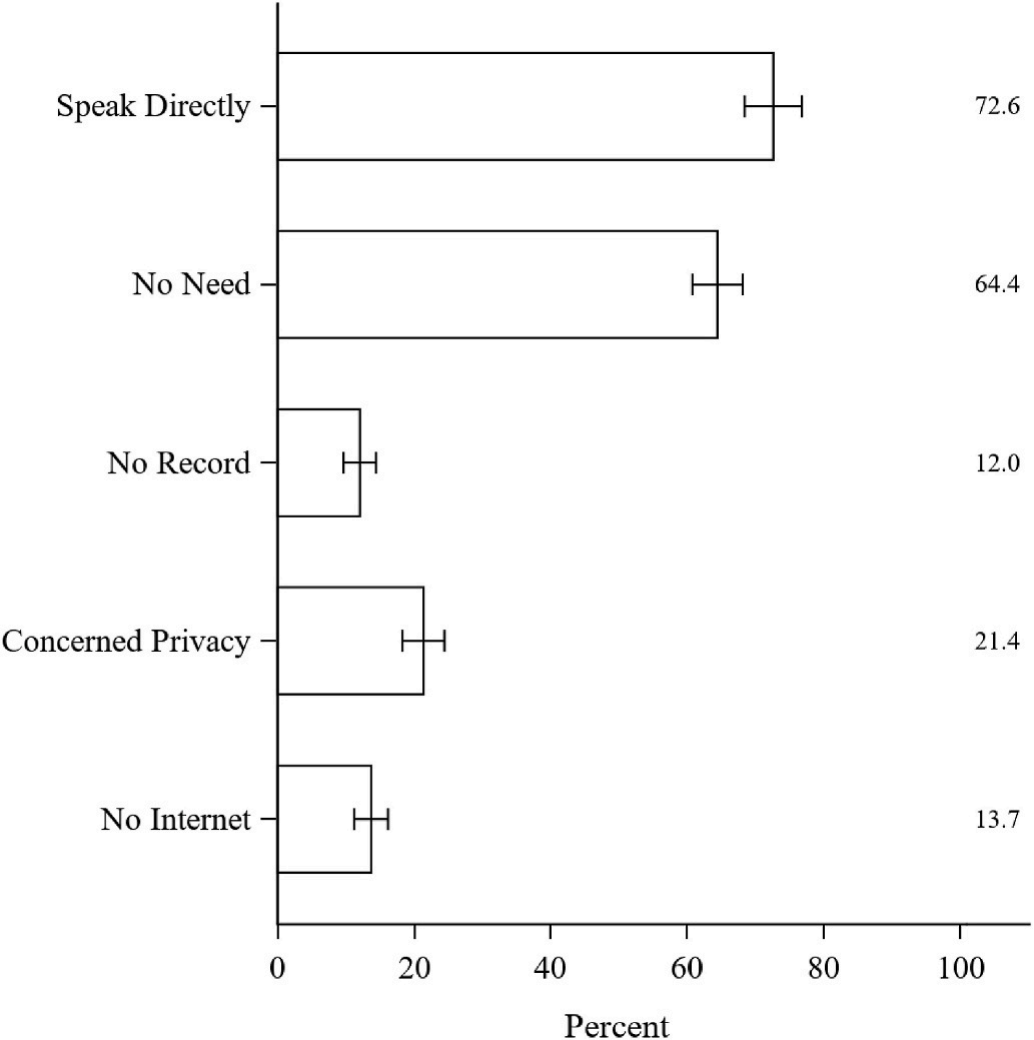
Percentages of five specific reasons that explain why respondents did not access their online medical records during the past 12 months.

Fig. 3 showed the proportions of respondents who answered one of five reasons for why they have no access to their online records during the past 12 months. The top two reasons were “prefer to speak to their health care provider directly” (72.58%, 95% CI: 68.38%–76.78%) and “did not have a need to use online medical record” (64.42%, 95% CI: 60.72%–68.12%). The findings of barriers from patients’ desire and emotions are consistent with prior work (Amante et al., 2014). The proportions of the other three reasons were considerably lower, which accounted for 12.02% for “do not have online medical record”, 21.36% for “Have concerned privacy”, and 13.68% for “Do not have access to the internet”.

## 4 Discussion

In this study, we analyzed The Health Information National Trends Survey (HINTS) data to observe the usage gap between EHR adoption and access for patients at geographical level. There is empirical evidence indicating a substantial expansion in the number of healthcare providers in the United States offering EHR portals for patient access (Office of Disease Prevention and Health Promotion, 2020; Office of the National Coordinator for Health Information Technology, 2022); however, only 30% of healthcare patients in the United States use their online portals (Johnson et al., 2020). The massive gap between patients who were being offered and who were actual accessing the portals and the barriers that prevent patients from accessing their EHR are the top concerns that bother not only the patients but our public health system. Our study attempted to use both statistical and spatial methods to address these concerns.

Prior studies focused on the socio-economic impacts on accessing EHR among patients, and they found that low income, minority, elderly, and uninsured population groups tend to have lower odds of using their EHR portals (Andrulis, 1998; Betancourt et al., 2003; Coffman et al., 2014; Hing & Burt, 2009; Krahn et al., 2006; Yeager et al., 2010). Our results are consistent with previous work, but we have more findings. By using the 2019–2020 HINTS data, we found that, of the total population, highly educated respondents were more likely to access online EHR portals. Additionally, people who were regularly looking for health information, insured and had regular providers tended to access their EHRs. Finally, our findings suggest that individuals residing in underserved regions, as well as those in urban areas characterized by disadvantaged socioeconomic statuses, exhibit a reduced likelihood of accessing online portals.

Geographical variation of EHR use gap was considerable at the national level and state level. The gap was much higher in Midwest and Northeastern States, except for Massachusetts and Washington D.C. areas. Southeast regions tend to have a relatively narrow gap than other States. Meanwhile, the West-Coast States, aka Hi-Tech States, and West South-Central division, such as Arizona and Texas, had better performance in adopting EHR portals for patients. Among the patients who offered EHR, those who living in Midwest or South regions had lower odds of accessing than other areas.

The geographic patterns of EHR portal use among patients in our study are consistent with prior works and reconfirmed that socioeconomic disparities are coincided with healthcare disparities, especially for accessing e-health services (Coffman et al., 2014; Health Information National Trends Survey; Hing & Burt, 2009; Munir & Boaden, 2001; Roblin et al., 2009; Rutten et al., 2012; Yeager et al., 2010). People in the Midwest, Southern regions, and Mountain rural areas are more likely to have greater socioeconomic inequality than other regions (Harper & Lynch, 2007; McGarvey et al., 2011; Snead & Cockerham, 2002). Therefore, populations residing in these underserved areas tend to face more considerable obstacles to e-healthcare.

Understanding the geographic pattern of current EHR usage and socio-economic barriers provides a way to improve the use of new technology to avoid the existing difficulties, especially in post-COVID19 era, such as mobile technologies or smartwatch, to help patients more comfortable accessing the EHR portal and monitoring their health conditions. Moreover, this information can be used in conveying public healthcare resources, educated people in varying geographical regions. Finally, healthcare providers, community social workers, local governments could use such data to improve their interpersonal communication, educated and trained social-health workers and implement strategies for targeted population groups who has no experience with e-health technology, or have no access to internet, or people who more concern about their health privacy.

Further research is needed to explore barriers in accessing EHR among patients in varying regions. Such research must take into consideration that patients in different regions may have different barriers for their accessing, including economics, environmental, cultural, social, and health-related barriers. We need to better understand the local barriers to provide a more effective way to increase EHR patient portals’ adoption and usage. Further research also needs to focus on if online portals are more beneficial for specific provider and patient populations, such as medical specialties or health conditions that require more communication between the provider and patient, but not necessarily face to face visits. Finally, future work should find ways to facilitate and influence portal adoption and support patient acceptance and use of portals, especially in the improvement of user experience, security, functionality, and user interface.

## 5 Limitations

Our study has several limitations. First, the survey design for HINTS is cross-sectional. Although we observed associations between demographic characteristics and patients’ use of 10.13039/100000081EHR, definitive conclusions cannot support a causal inference. Second, the response rate for the selected survey was approximately 32%, which may lead to selection and estimation bias. We suggest that local or micro-level studies are conducted to validate these findings, which could give a better, detailed interpretation of EHR access availability and use. Third, the survey information only collected data through a relatively short period, so it may not grasp the rapid change of EHR patient portal usage in the following periods. Also, since the HINS 5 cycle 4 dataset was collected during the COVID-19 period, the effect of the pandemic which may greatly affect the analysis is not examined in the study. Finally, our study cannot address all impact factors on the portal use, and several possible variables, such as financial constraints, insufficient technological support, safety considerations, and implementation challenges on 10.13039/100000081EHR adoption and access, may contribute to additional explanations for the association that was not included in our data.

## 6 Conclusions

Despite the widespread adoption of EHR systems in recent years, and prominent benefits that EHR patient portal brought about to every participant of the healthcare system, the enormous gaps of using patient portals still exist not between patients who were offered and who had access, but among varying regions. Our study, consistent with prior findings, indicates that (1) patients with suboptimal demographic profiles residing in underserved regions (both in deprived urban and non-urban areas) exhibit a heightened proclivity to encounter disparities in both the provision of physical healthcare services and accessibility to virtual health information. These inequities, in turn, contribute to the degradation of their health status and overall quality of life; (2) Individuals residing in the Midwest, Southern, and Mountain areas exhibit a higher propensity for encountering heightened challenges in accessing EHR when compared to populations in other geographic regions; (3) female, uninsured, less educated, lack of E-health resource, no regular provider, no insurance or living in rural areas or deprived urban areas patients are more likely having barriers to access their patient portals. To ameliorate such disparities, efforts of education and encouragement from both healthcare providers and policymakers can further facilitate the adoption rate of EHR patient portal by using the health information technologies to improve health care and health outcomes. Furthermore, to maximize the benefits of EHR patient portal to meet the needs of all patients, especially those who are traditionally underserved by the healthcare system, future work should focus on the accessibility and use of eHealth resources for target population groups. Finally, future research should understand and explore the preferences of using eHealth technologies not only by specialty among providers but for patients with different demographic backgrounds and health conditions. Such studies would be beneficial to unveil the deeper causes of low-use groups and regions.

## Ethical Statement for Solid State Ionics

Hereby, I, Qinglin Hu, consciously assure that for the manuscript “Examining the Existing Usage Gap of Electronic Health Records in The United States: A Study of National Health Survey” the following is fulfilled:

1) This material is the authors' own original work, which has not been previously published elsewhere.
2) The paper is not currently being considered for publication elsewhere.
3) The paper reflects the authors' own research and analysis in a truthful and complete manner.
4) The paper properly credits the meaningful contributions of co-authors and co-researchers.
5) The results are appropriately placed in the context of prior and existing research.
6) All sources used are properly disclosed (correct citation). Literally copying of text must be indicated as such by using quotation marks and giving proper reference.
7) All authors have been personally and actively involved in substantial work leading to the paper, and will take public responsibility for its content.

The violation of the Ethical Statement rules may result in severe consequences.

To verify originality, your article may be checked by the originality detection software iThenticate. See also http://www.elsevier.com/editors/plagdetect.

I agree with the above statements and declare that this submission follows the policies of Solid State Ionics as outlined in the Guide for Authors and in the Ethical Statement.

## Authors’ contributions

QH and JH designed the study, obtained the data, and drafted the manuscript. QH, YY and XY preformed the analysis and interpreted the results. JH and JP critically reviewed and edited the manuscript. All authors read and approved the final manuscript.

## Declaration of competing interest

No potential conflict of interest was reported by the author(s).

## Figures and Tables

**Table 1:** Demographic characteristics of the nationally representative sample

Variable	Level	Total (n = 5235) n(%)	Access to Online EHR (n = 3402) n(%)	No Access to Online EHR (n = 1833) n(%)	Chi-square test p value

Gender	Male	1,945 (43.0)	1,255 (41.8)	690 (45.3)	0.2192
	Female	3,202 (57.0)	2,097 (58.2)	1,105 (54.7)	

Education	Less than or completed high school	852 (21.4)	396 (16.8)	456 (30.3)	<.0001
	Some college	1,518 (42.3)	937 (41.4)	581 (44.0)	
	College graduate or higher	2,761 (36.3)	2,013 (41.8)	748 (25.7)	

Age groups	18–24	672 (23.3)	489 (24.8)	183 (20.3)	0.0013
	25–44	1,021 (26.7)	713 (27.8)	308 (24.5)	
	45–64	1,635 (30.8)	1,083 (30.2)	552 (31.9)	
	65–74	1,202 (12.5)	771 (11.9)	431 (13.6)	
	75+	600 (6.8)	296 (5.3)	304 (9.7)	

Race	Hispanic	595 (12.9)	357 (10.9)	238 (16.8)	0.0060
	NH-White	3,289 (69.1)	2,229 (71.3)	1,060 (64.7)	
	NH Black	609 (9.9)	374 (9.0)	235 (11.6)	
	Asian/others	390 (8.2)	268 (8.8)	122 (6.9)	

Urban/Rural	Urban	4,720 (88.6)	3,109 (90.1)	1,611 (85.6)	0.0095
	Non-urban	515 (11.4)	293 (9.9)	222 (14.4)	

Household income	Less than $20,000	571 (10.9)	271 (9.2)	300 (14.5)	0.0003
	$20,000 - $50,000	1,122 (22.1)	661 (20.3)	461 (25.8)	
	$50,000 - $75,000	904 (18.3)	593 (18.3)	311 (18.4)	
	$75,000+	2,155 (48.6)	1,592 (52.2)	563 (41.3)	

General health	Excellent/Very good	2,651 (52.2)	1,785 (53.7)	866 (49.4)	0.1450
	Good	1,825 (34.7)	1,177 (34.4)	648 (35.3)	
	Fair/poor	711 (13.1)	407 (12.0)	304 (15.3)	

Look for heath info.	Yes	4,219 (81.6)	3,046 (89.3)	1,173 (66.8)	<.0001
	No	970 (18.4)	332 (10.7)	638 (33.2)	

Has health insurance	Yes	5,069 (95.3)	3,321 (96.6)	1,748 (92.9)	0.0255
	No	132 (4.7)	67 (3.4)	65 (7.1)	

Has regular provider	Yes	4,055 (74.5)	2,758 (78.8)	1,297 (66.2)	<.0001
	No	1,120 (25.5)	611 (21.2)	509 (33.8)	

**Table 2:** Global tests for the adjusted logistic regression model

Test	F Value	Num DF	Den DF	p value

Likelihood Ratio	24.31	13.9304	682.59	Ref. *P < .001*

Score	11.66	19	49	Ref. *P < .001*

Wald	11.88	19	49	Ref. *P < .001*

NOTE: Second-order Rao-Scott design correction 0.3639 applied to the Likelihood Ratio test.

**Table 3:** Odds ratio and 95% confidence intervals of multiple logistic regression models

Variable	Level	Crude ORs odds ratio(95% CI) ^1^	Adjusted ORs odds ratio(95% CI) ^2^	Adjusted log(ORs) Log odds ratio (95% CI)

Gender	Male	Ref. *P = .113*	Ref. *P = .248*	Ref. *P = .248*
	Female	1.21 (0.95, 1.55)	1.18 (0.89, 1.58)	0.17 (−0.12, 0.46)

Education	Less than or completed high school	Ref. *P < .001*	Ref. *P < .001*	Ref. *P < .001*
	Some college	1.82 (1.27,2.62)	1.48 (1.04,2.11)	0.39 (0.04, 0.75)
	College graduate or higher	3.01 (2.29,3.97)	2.01 (1.44,2.80)	0.70 (0.36, 1.03)

Age group	18–24	Ref. *P = .02*	Ref. *P = .05*	Ref. *P = .05*
	25–44	1.00 (0.66,1.51)	1.03 (0.65,1.64)	0.03 (−0.43, 0.49)
	45–64	0.90 (0.63,1.30)	0.84 (0.54,1.30)	−0.17 (−0.62, 0.26)
	65–74	0.88 (0.60,1.28)	0.79 (0.50,1.25)	−0.24 (−0.69, 0.22)
	75+	0.55 (0.36,0.85)	0.60 (0.38,0.96)	−0.51 (−0.97, −0.04)

Race	Hispanic	Ref. *P = .004*	Ref. *P = .20*	Ref. *P = .20*
	NH-White	1.76 (1.19,2.61)	1.65 (0.98,2.76)	0.50 (−0.02, 1.02)
	NH Black	1.18 (0.71,1.95)	1.31 (0.72,2.37)	0.27 (−0.33, 0.86)
	Asian/others	2.15 (1.25,3.71)	1.89 (0.96,3.76)	0.64 (−0.04, 1.32)

Urban/Non-urban	Urban	Ref. *P = .03*	Ref. *P = .01*	Ref. *P = .01*
	Non-urban	0.66 (0.46,0.95)	0.60 (0.41,0.89)	−0.51(-0.89, −0.12)

Household income	Less than $20,000	Ref. *P = .007*	Ref. *P = .81*	Ref. *P = .81*
	$20,000 - $50,000	1.26 (0.79,2.01)	1.24 (0.79,1.96)	0.22 (−0.24, 0.67)
	$50,000 - $75,000	1.47 (0.90,2.38)	1.14 (0.71,1.83)	0.13 (−0.34, 0.60)
	$75,000+	1.91 (1.27,2.85)	1.13 (0.76,1.67)	0.12 (−0.27, 0.51)

General health	Excellent/Very good	Ref. *P = .38*	Ref. *P = .74*	Ref. *P = .74*
	Good	0.92 (0.70,1.20)	1.08 (0.80,1.46)	0.08 (−0.22, 0.38)
	Fair/poor	0.75 (0.49,1.14)	0.97 (0.63,1.50)	−0.03(-0.46, 0.41)

Look for health	No	Ref. *P < .001*	Ref. *P < .001*	Ref. *P < .001*
	Yes	4.10 (2.96,5.69)	3.51 (2.49,4.94)	1.26 (0.91, 1.60)

Has health insurance	No	Ref. *P = .010*	Ref. *P = .04*	Ref. *P = .04*
	Yes	2.77 (1.30,5.93)	2.38 (1.05,5.43)	0.87 (0.05, 1.69)

Has regular provider	No	Ref. *P < .001*	Ref. *P < .001*	Ref. *P < .001*
	Yes	1.96 (1.50,2.56)	2.10 (1.51,2.92)	0.74 (0.41, 1.07)

^1^Crude ORs.

^2^Adjusted ORs for.

## Data Availability

The authors do not have permission to share data.
